# Research on the Manufacturing Process and Heat Transfer Performance of Ultra-Thin Heat Pipes: A Review

**DOI:** 10.3390/ma15155459

**Published:** 2022-08-08

**Authors:** Liuyang Duan, Hang Li, Jinguang Du, Kun Liu, Wenbin He

**Affiliations:** Henan Provincial Key Laboratory of Intelligent Manufacturing of Mechanical Equipment, Zhengzhou University of Light Industry, Zhengzhou 450002, China

**Keywords:** ultra-thin heat pipe, manufacturing process, heat transfer performance, heat transfer mechanism

## Abstract

This paper reviews the manufacturing process of ultra-thin heat pipes and the latest process technologies in detail, focusing on the progress of the shape, structure, and heat transfer mechanism of the wick. The effects of the filling rate and tilt angle on the heat transfer performance of the ultra-thin heat pipe, as well as the material selection of ultra-thin heat pipes, is sorted out, and the surface modification technology is analyzed. Besides, the optimal design based on heat pipes is discussed. Spiral woven mesh wick and multi-size composite wick have significant advantages in the field of ultra-thin heat pipe heat transfer, and comprehensive surface modification technology has huge potential. Finally, an outlook on future scientific research in the field of ultra-thin heat pipes is proposed.

## 1. Introduction

With the development of microelectronics technology, electronic devices are gradually developing towards integration, lightness, and high performance. High performance results in high power consumption. Meanwhile, integration and thinness lead to the continuous reduction in heat dissipation space. These factors can lead to the rapid growth of heat flux, which makes the cooling system face great challenges. Not only that but with the advent of the 5G network communication era, the high heat flow density caused by the high-speed data transfer and processing of electronic chips can also bring more severe challenges. Research data shows that the heat flow density of high-end electronics has reached more than 200 W/cm^2^ [1]. The high heat flow density causes rapid heat up of the chip surface, which seriously affects the reliability and lifetime on electronic chips [2,3,4]. The high heat flow density, caused by the imbalance between heat generation and heat dissipation, is the most important reason that hinders the normal operation of electronic devices [5,6]. In addition, uneven chip surface temperature is also an adverse factor that leads to electronic device failure [7]. Therefore, the ways to solve the heat dissipation problem of electronic devices and the development of high heat dissipation performance devices have become a hotspot and difficult point of research, so it is urgent to find a suitable solution.

**Figure 1:**
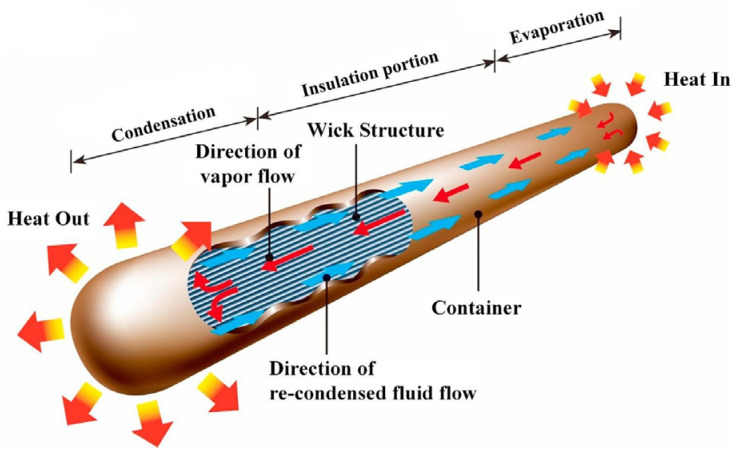
Schematic diagram of the heat pipe principle [8].

As shown in Figure 1 [8], as a passive heat dissipation component uses the vapor–liquid phase transformation of the working fluid to transfer heat, heat pipes have the advantages of excellent transient response, super high thermal conductivity, long life, and good isothermal performance. Therefore, it can effectively dissipate the heat flow and is widely used in the cooling system of electronic devices, which is an optimal solution for the heat dissipation of electronic devices [9,10]. Under the premise of ensuring high heat transfer performance, in order to cooperate with the development of electronic equipment, the heat pipe has been continuously developed in the aspects of lightness and thinness, and it gradually evolved into an ultra-thin heat pipe with a flattened thickness of less than 2 mm.

**Figure 2:**
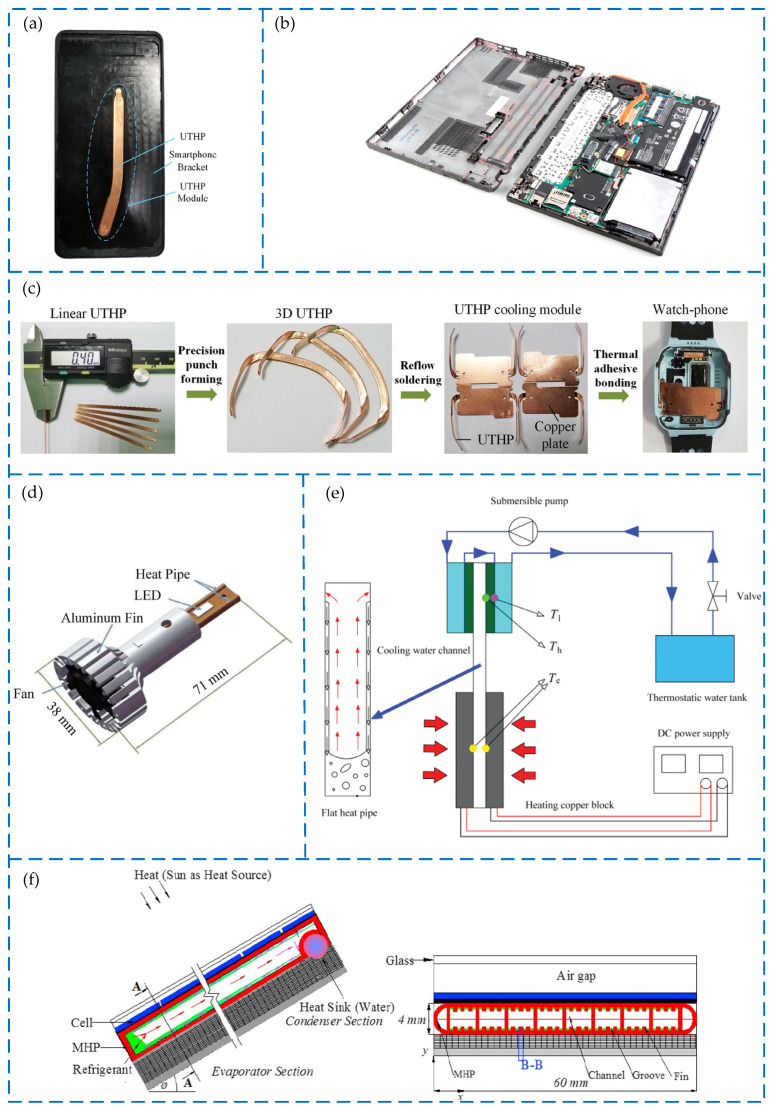
Applications of UTHPs: (**a**) smart phone [11]; (**b**) laptop [12]; (**c**) smart watch [13]; (**d**) LED [14]; (**e**) solar thermoelectric generator [16]; (**f**) solar photovoltaic thermal system [17].

As shown in Figure 2 [11,12,13,14,15,16,17], ultra-thin heat pipes have good application prospects because of their excellent heat dissipation performance and flexible structure in electronic mobile devices, high-power LEDs, and solar installations. In order to optimize the heat dissipation structure of mobile phones and to solve the problems of insufficient heat dissipation capacity, thick stacking, and large mass of traditional graphite or copper sheets, Zhou et al. [11] designed an ultra-thin heat pipe, comparing it to copper sheets, and found that the ultra-thin heat pipe not only increases the maximum heat dissipation power by 28.57~42.86% but also reduces the weight of the heat dissipation structure by 64.51%. Zhou et al. [12] applied a 1 mm ultra-thin heat pipe to a notebook computer and found that the maximum heat flux of the ultra-thin heat pipe on the notebook computer can reach 15 W under natural convection. It can dissipate up to 25 W of heat flux under the condition of forced air cooling assisted by the fan [12]. Zhou et al. [13] used a welding technology to embed the ultra-thin heat pipe in the smart watch, and tested the heat dissipation performance, with reference to the copper block of the same size. The results showed that the thermal resistance of the ultra-thin heat pipe is 2.63 °C/W; the maximum heat flux is 0.9 W; the data is better than the copper block; it also has a faster heat dissipation speed [13]. Tang et al. [14] constructed a heat sink platform based on heat pipes for high-power LED lamps, which was applied to automobiles, and the test data showed that the minimum thermal resistance is 1.86 °C/W and the maximum thermal resistance is less than 2.17 °C/W. Ji et al. [15] tested the high-temperature heat pipe (HTHP) by simulating solar energy with a xenon lamp module. When the pipe wall temperature reaches as high as 700 °C, the equivalent thermal resistance is less than 0.01 KW^−1^, and the contact thermal resistance is almost zero [15]. As shown in Figure 2e [16], Zhao et al. cleverly applied the heat pipe to the solar thermoelectric generator. Under the heating power of 60 W, the peak power output could reach 0.94 W, and the conversion efficiency could reach 1.57% [16]. Modjinou et al. [17] adopted an aluminum-based micro-channel heat pipe (MHP) to assist the solar photovoltaic thermal system, as shown in Figure 2f [17], and the maximum instantaneous thermal efficiency reaches 54%.

**Figure 3:**
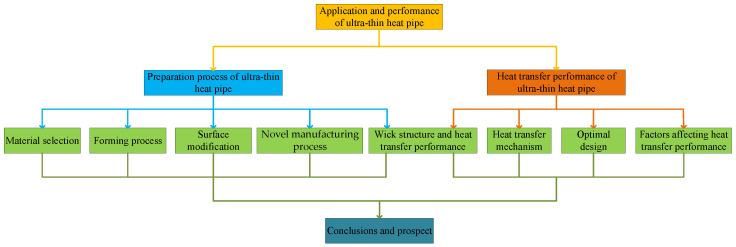
The framework structure of this paper.

As shown in Figure 3, this paper reviews the manufacturing process and heat transfer performance of ultra-thin heat pipe. First, this paper introduces the selection of materials and the manufacturing process of wick. Then, the influence of surface modification and wick structure on heat transfer performance were comprehensively analyzed, the latest research results of phase change heat transfer of ultra-thin heat pipes were summarized, the heat transfer mechanism and phenomena produced in the operation process of the heat pipe were explained, theoretically, and the factors affecting heat transfer performance and optimal design were analyzed. Finally, the future research direction of the ultra-thin heat pipe was prospected.

## 2. Manufacturing Process for Ultra-Thin Heat Pipes

**Figure 4:**
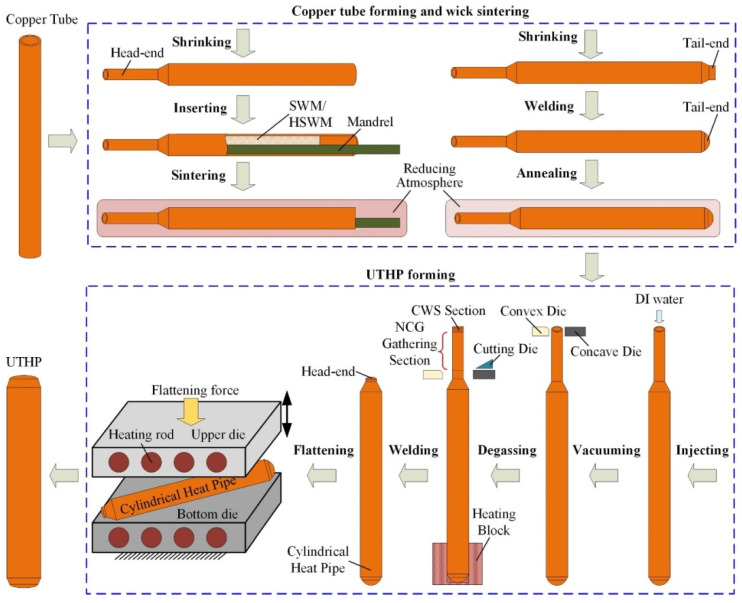
Flow chart of the main manufacturing process of ultra-thin heat pipes [18].

The main manufacturing process of the ultra-thin heat pipe is shown in Figure 4 [18]. Compared with the other heat transfer elements, the manufacturing process of ultra-thin heat pipes has fewer and simpler steps, as well as lower manufacturing costs. Although the manufacturing processes of ultra-thin heat pipes are generally similar, there are local differences in some processes, such as the selection and matching of shell materials and wick materials, the process of shrinking the shell, and the welding methods. This paper mainly focuses on the process of material selection, wick manufacturing, adding the super hydrophilic treatment, and new manufacturing process.

### 2.1. Materials Selection of Ultra-Thin Heat Pipes

Different materials have different thermal conductivity, and the selection of heat transfer materials significantly impacts the heat transfer performance of ultra-thin heat pipes. The materials used to prepare ultra-thin heat pipes mainly contain the shell material, the wick material, the mandrel material, the type of working fluid, etc. The selection of the materials for each working part should be comprehensively considered according to the thermal conductivity, machining performance, and compatibility between materials. The material and structure of wicks are complex, so they are explained and analyzed separately in Section 2.2.

The pipe shell material of ultra-thin heat pipe is generally selected from copper, aluminum, stainless steel, and other materials. Whether it is compatible with the working fluid and the wick is the primary consideration in the selection of pipes. Yuan et al. [19] developed an aluminum-based micro-grooved heat pipe using acetone as the working fluid, and the minimum thermal resistance can reach 0.029 KW^−1^. However, aluminum is easily corroded by acid and alkali during long-term use, and the welding seal will also weaken the corrosion resistance of the aluminum pipe. At the concentration of 0.125 mol L^−1^NaOH, the maximum resistance time of the heat pipe is only 110 min [19]. Due to the incompatibility of aluminum and water, under the heating conditions of heat pipe operation, aluminum reacts with water to generate non-condensable hydrogen, so the water cannot be selected as the working fluid when aluminum is selected as the shell material [20]. The non-condensable gas will hinder the gas–liquid circulation of the heat pipe, and the hindering effect is more obvious at low heating power [20]. Copper heat pipes and stainless steel heat pipes generally use water, methanol, acetone, and ammonia as working fluids because of their ideal compatibility. Zhang et al. [21] designed a heat pipe with a stainless steel pipe and a stainless steel powder wick. Compared with copper, it has excellent mechanical properties and has wider adaptability to the environment [21]. Its maximum permeability is 1.299 × 10^−11^ m^2^ [21]. The heat transfer effect is limited [21]. The advantages and disadvantages of heat pipes of different pipe materials and their application fields are shown in Table 1 [11,12,13,14,15,16,17,19,21,22,23,24]. Based on compatibility, the material with good thermal conductivity, long service life, good stability, low economic cost, good process performance, and easy processing is selected on a merit basis.

The flat-plate ultra-thin heat pipe is generally chosen as flat pipe; while for flat ultra-thin heat pipe used more widely, the cylindrical pipe is usually chosen. Currently, the copper is most commonly used as the heat pipe shell material, and it has good compatibility and wettability with the working fluid deionized water [25,26]. Copper is divided into oxygen-free copper, phosphorus deoxidized copper, common copper, silver copper, etc., in which the phosphorus deoxidized copper is generally chosen, in the TP1, as the shell material [27,28]. It does not occur with “hydrogen embrittlement” and brittleness at high temperatures, which affects the pipe processing [27,28]. In addition, TP1 has lower phosphorus content than TP2, so TP1 has better electrical and thermal conductivity. Table 2 [27,28] gives the properties of TP1 optical pipes.

Whether powder sintering or mesh sintering, the wick needs to be fixed in the pipe using a mandrel first. The mandrel material is usually stainless steel (310S) or ceramic (boron nitride, BN) [28]. In fabrication processing, the stainless steel mandrel is sprayed with boron carbide, as a release agent, in order to facilitate the removal of the mandrel after sintering and to increase the number of uses [28]. This release agent attaches to the wick during the sintering process, which interferes with the return flow of the working fluid. Ceramic mandrels do not require treatment [28]. In terms of reuse, the 310S mandrels can only be reused 5~7 times because of them being prone to rusting, while ceramic mandrels can be reused more than 30 times [27]. In terms of the performance of the wicks, the performance of wicks using ceramic mandrels for solid-phase sintering is, mostly, slightly better than that of the wicks using 310S mandrels [27]. Given the cost, 310S mandrels are less expensive, while ceramic mandrels are more expensive and fragile [27]. In summary, 310S mandrels are suitable for small-scale experimental studies, while ceramic mandrels are suitable for large-scale production and applications.

**Figure 5:**
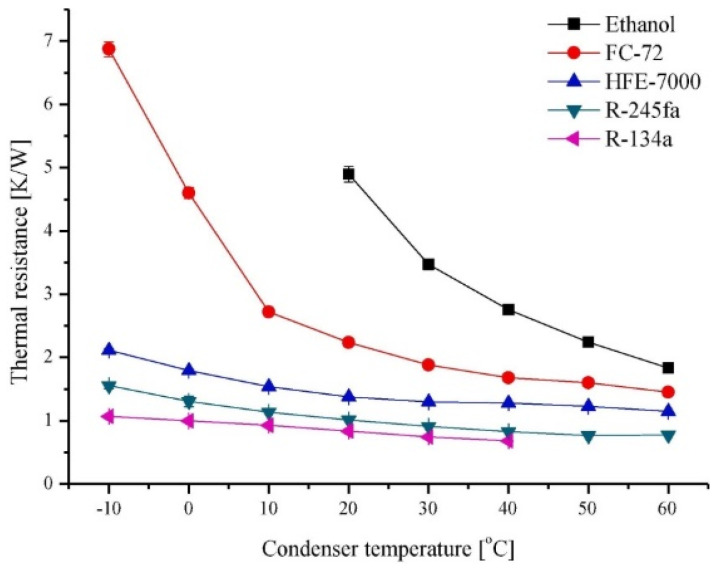
Comparison of the thermal resistance of MPHP for different working fluids [33].

The thermal properties of the working fluid and the heat transfer performance of the ultra-thin heat pipe are also closely related, and the selection of a suitable working fluid is critical, as shown in Table 3 [29,30,31]. To pursue a higher performance ultra-thin heat pipe, researchers have searched for numerous working fluids to investigate. Chao et al. [32] compared the heat transfer characteristics of three different working fluids (acetone, ethanol, and methanol) through experiments and found that acetone is the most suitable working fluid under the lower heating power, while under the condition of higher heating power, ethanol has better heat transfer performance. Based on the previous work (Figure 5 [33]), Juno Kim and Sung Jin Kim [33] selected five working fluids (ethanol, FC-72, HFE-7000, R-245fa, and R-134a), and each micro-pulsating heat pipe (MPHP) was filled with different working fluids, at room temperature, with the input power of Q = 15 W and the filling rate being 50%. The corresponding thermal resistance values were obtained by adjusting the different condenser temperatures [33]. The experimental data showed that the micro-pulsating heat pipe with R-134a had the lowest thermal resistance, which was ten times smaller than the MPHP with FC-72 [33]. In addition, the concept of a quality factor is proposed to facilitate researchers to select the optimal working fluid, with the lowest thermal resistance in pulsating heat pipes, more easily [33]. Khan et al. [34] constructed a hybrid nanofluid based on ethylene glycol with high thermal conductivity and excellent heat transfer potential. In addition, Khan et al. [35] also built nanoliquid films on cylinders to achieve efficient heat transfer through spraying technology.

In summary, as a key factor directly affecting the heat transfer performance, the material selection needs to be considered from various aspects, such as material compatibility, mechanical strength, thermal conductivity, material cost, etc. For example, TP1 of smooth inner wall is preferred as the shell material. However, it is also necessary to take into account the actual situation to match the most suitable material in the actual application process.

### 2.2. Wick Structure and Heat Transfer Performance

As the power element of an ultra-thin heat pipe, the wick needs to provide a high capillary force to facilitate the circulation of the working fluid for heat transfer, on the other hand the material should have good thermal conductivity. The types of wicks are very complicated, but the materials used to prepare the wicks are relatively simple. For most wicks, copper and some metals, such as stainless steel [36], nickel [37], or non-metallic ceramics [38] and carbon fiber [39], are used as the preparation material of wicks.

The raw material for wicks is generally in the form of powder or wire (fiber), and the different material forms imply different methods of forming processes. At present, most of the raw materials for wicks are in the form of copper powder, and a small number of copper wires (fibers) have been reported. As a new type of wick structure, copper wire sintered braided mesh core usually uses 0.04 mm or 0.05 mm diameter copper wire as the preparation material [11,18,28,40,41]. Compared with the cutting method, the copper wire prepared by the wire drawing method has a smooth surface, uniform and accurate diameter, low production cost, and short preparation period [28]. However, the wick has poor hydrophilicity and low capillary capacity, which affects the heat transfer capacity [28]. Therefore, the wick made of copper wire needs to be super-hydrophilized [28].

An ultra-thin heat pipe usually consists of a pipe shell, a wick, and a working fluid. As the core part, the shape and structure of the wick are critical to the performance of the ultra-thin heat pipe. The wick is used to provide power to the inside of the ultra-thin heat pipe through capillary action, which leads to the formation of a thermal cycle inside to transfer heat energy. According to the structure, it is divided into sintered wick, grooved wick, and composite wick [42]. Among them, powder sintered wicks are the most widely used, as wicks rely on their cost-effectiveness [40]. In contrast, the traditional screen sintered wicks have a large internal pressure drop, a low capillary pressure of wick, high thermal resistance of the contact with the shell, and a poor gravity resistance of the grooved wicks, which have a large fluctuation in performance under weightlessness and when overweight, so the application rate is relatively low [43,44,45,46,47]. According to the data in Table 4 [43,44,45,46,47], it can be concluded that the maximum heat transfer capacity of a powder type wick is 30–70% higher than that of other types of wicks with the same thickness. In addition, the advantage of heat transfer performance is especially prominent, and the resistance to gravity is strong [40].

**Figure 6:**
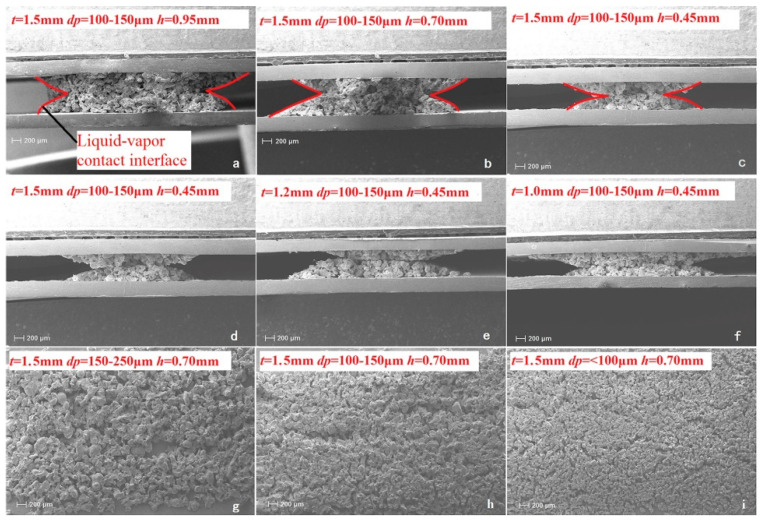
SEM photographs of the wick structure with different parameters: *t* is the heat pipe squash thickness, *dp* is the copper powder particle size, and *h* is the maximum thickness of the absorbent wick [48].

With the development of heat pipe in fineness and the continuous exploration of heat pipe performance, it is found that there are problems in the heat transfer performance and stability of powder sintered heat pipe, which makes it difficult to ensure the high load operation of high heat flux equipment when the flattening thickness is small [48]. As shown in Figure 6 [48], with the reduction in the flattening thickness, the partial deformation of the powder sintered wick structure affects the porosity of the wick [48]. The change of porosity directly affects the heat transfer performance of the heat pipe. The powder-sintered wick structure easily to produces cracks or even falls off when the thickness of the flattening is thin, which will also affect the heat transfer performance and stability of the heat pipe to different degrees [48]. Li et al. [49] developed a new ultra-thin heat pipe for cell phone cooling systems, which adopts a spiral woven mesh wick structure with good heat transfer and low manufacturing cost, and they began to gradually replace powder sintered heat pipes in the field of ultra-thin heat pipes. However, the spiral woven wire mesh wick has the same problem of low capillary capacity as the traditional mesh wick [50]. To solve this problem, Tang et al. [50] introduced the chemical deposition method by depositing the woven copper wire mesh into a 250 mL mixture of 10 mL/L NaOH and 0.5 mol/L K_2_S_2_O_8_, which significantly improved the capillary capacity of the spiral woven wire mesh wick. In addition, the heat transfer performance was tested by varying the sintering temperature and chemical deposition time, and the experimental data showed that the best heat transfer performance was obtained from the group with the sintering temperature of 500 °C and chemical deposition of 15 min for the wick [50].

**Figure 7:**
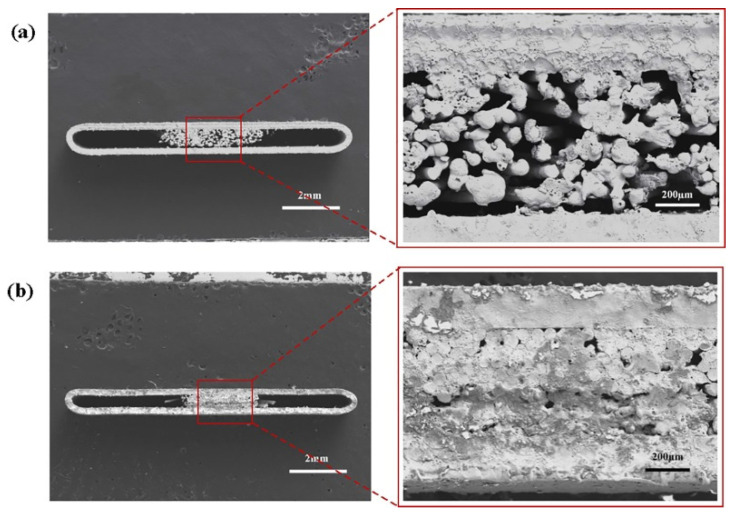
SEM image of a spiral braided mesh wick ultra-thin heat pipe [40]. (**a**) *t* *=* 1.0 mm (**b**) *t* = 0.8 mm.

The spiral woven mesh is made by the interlaced weaving of the knitting machine, which is very suitable as wick structure of ultra-thin heat pipe [50]. It has the advantages of compact structure, regular pore, good flexibility, a simple manufacturing process, and low manufacturing cost. The braiding starts by combining several copper wires into one strand, rotating multiple strands on the cylindrical mandrel through the braiding machine, and interweaving them into a circular spiral braided mesh structure [50]. The double-layer spiral woven mesh is then placed in the center of the machine rotating wheel and woven again to form a circular double-layer structure with close contact [50]. Tang et al. [40] studied the effect of different heat pipe thicknesses on the spiral woven mesh type ultra-thin heat pipe, as shown in Figure 7 [40]. Most of the pores disappear when the thickness of the heat pipe decreases to 0.8 mm [40]. The structure of 0.05 mm copper fiber spiral braided mesh wick was seriously damaged, and the heat pipe was basically in a failure state [40]. Similarly, Zhou et al. [51] also explored in the spiral woven mesh type ultra-thin heat pipe, and it can be seen, from Table 5, that the maximum heat transfer capacity of the ultra-thin heat pipe is only 3.6 W when the thickness is 0.4 mm. It is difficult to solve the problem of heat pipe failure, when the thickness is low, by reducing or increasing the diameter of copper wire. When the diameter of the copper wire is too small, the mechanical strength of the wick is insufficient. In addition, the structure is easily damaged when the phase change flattens or bends. However, the diameter of copper wire should not be too large because it is limited by the thickness of heat pipe.

**Figure 8:**
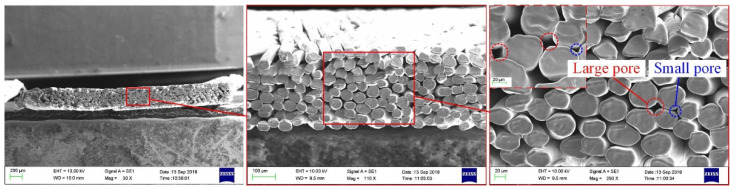
SEM images of composite woven mesh wick [18].

As shown in Table 5 [11,40,41,51,52], in pursuit of a heat pipe with thinner thickness and better heat transfer performance, many scholars have performed further studies on wire mesh braiding structure. Yang et al. [52] designed a composite braided mesh wick (B_4_) with a double-layer structure, which was made by wrapping 0.1 mm copper wire with 0.05 mm copper wire. Compared with traditional spiral braided mesh wick (B3), the overall heat transfer performance improved by 32.5% [53]. Zhou et al. [41] designed double-layer spiral woven mesh wicks (C_1_, C_2_) with 0.04 mm and 0.05 mm copper wires, according to the strand structure. In addition, a single-hole, three-layer spiral woven mesh wick (C_3_), consisting of only 0.04 mm copper wires, was also designed [41]. The results showed that the maximum heat transfer capacity of the three-layer mesh wick C_3_ was up to 26 W, and the minimum thermal resistance of C_2_ was at 0.101 °C/W [41]. The maximum heat transfer capacity of C_2_ was 7.70% lower than that of C_3_, but the thermal resistance was reduced by 6.32–25.90% [41]. In addition, the copper wire is reduced by 22%, and C_2_ is the best from both the cost and performance perspectives, which indicates that the heat transfer performance can be effectively improved by increasing the number of layers, but the comprehensive performance of the double braided mesh wick is the best [41]. To further explore the potential performance of the double-weave spiral mesh wicks with double holes, Zhou et al. [18] also designed eight types of double-weave spiral mesh wicks using 0.04 mm and 0.05 mm copper wires with different ratios of strands, and then, they tested the performance of heat transfer separately. The total thermal resistance was reduced by 27.53–42.92% compared to the conventional woven mesh wick. A larger pore space is formed between copper wires of different diameters in the hybrid weave, and a relatively smaller pore space is formed by copper wires of the same diameter (Figure 8 [18]). The larger pore space can improve the permeability of the wick, and the smaller pore space can enhance the capillary capacity to accelerate the thermal circulation, as well as reduce the overall size of the wick to leave more space for the steam, as well as liquid channels, inside the heat pipe [18]. The above study proves that the multi-size pore spiral woven mesh wick, prepared by mixing copper wires, can effectively improve the heat transfer performance of the ultra-thin heat pipe. According to the test data [11,40,41,51,52], it can be found that B_4_ is better, and the thickness of the ultra-thin heat pipe (B_4_) is thinner with the same maximum heat transfer capacity.

In the above studies, the toroidal spiral woven mesh was selected for wick structure, which squeezes too tightly and seriously affects the heat transfer during the phase change flattening process. In order to meet the requirement of 0.4 mm thickness of the smartphone heat sink system, Zhou et al. [11] designed a new ribbon spiral braided mesh wick, using 0.04 mm copper wire, consisting of 17 strands of copper wire, and they set three different wicks (A_1_, A_2_, and A_3_) by changing the number of copper wires per share to conduct performance tests in an ultra-thin heat pipe with a thickness of 0.4 mm. The experimental data shows that the A_3_ with 11 copper wires per share has the best heat transfer performance, with a maximum heat transfer capacity of 5.25 W [11]. However, A_2_ with 10 copper wires per share is more suitable if production cost is taken into account, and the maximum heat transfer capacity can still reach 5 W with 17 fewer wicks [11].

As shown in Table 5 [11,40,41,51,52], by comparing B_1_, B_2_, D_1_, and D_2_, it is found that the effect of chemical oxidation on heat transfer performance increases with the increase in heat pipe thickness, and the maximum heat transfer capacity increases to 45% when the thickness of heat pipe is 1.2 mm. By analyzing the data of five types of wicks from C_1_ to C_5,_ it can be seen that the number of layers, strand structure, number of strands, and the total number of copper wires all affect the heat transfer performance to different degrees [54]. When the thickness of the ultra-thin heat pipe is 1 mm, the maximum heat transfer capacity is improved by about 17.65% of B_1_ and B_2_, which can prove that the heat transfer performance can be increased by chemical oxidation [40]. The comparison of B_2_, B_4_, and the copper powder wick in Table 5 [11,40,41,51,52] conveyed that the maximum heat transfer capacity was close to the same, but in the comprehensive multifactor consideration, the manufacturing process of powder wick is more complicated, and the manufacturing yield is lower. Therefore, the spiral woven mesh wick is more suitable. In the ultra-thin heat pipe, with thickness of 1.1~1.2 mm, the maximum heat transfer capacity is 29 W [40], which is a significant improvement in heat transfer performance compared with the above-mentioned lower thickness heat pipes, and it indicates that the heat pipe thickness is an important factor affecting the heat transfer of ultra-thin heat pipes. When the thickness of the ultra-thin heat pipe is 0.4 mm, the volume in the cavity is severely reduced, and the heat transfer performance of the wick decreases tremendously. The performance requirements in all aspects are more stringent, and the ribbon spiral braided wick is the most optimal choice at present. There are still gaps concerning the total number of copper wires, strands, and multi-size pores. The field of heat pipes with 0.4 mm thickness is yet to be developed, and the numerical model regarding the ultimate ultra-thin thickness (the thickness less than 4 mm) needs to be developed separately. The volume of wick and the cavity space need to be adjusted for the optimal ratio, and the heat pipe shell and wick need excellent mechanical strength, in the phase change, when flattening to the ultimate thickness. Different experimentalists have different data measurement methods and instruments. The internal factors and external environmental factors of the heat pipe will differ, which need to be further verified, mutually, through theoretical analysis and experimental simulations.

**Table 5:** Structural parameters and maximum heat transfer capacity of spiral braided mesh wick ultra-thin heat pipe.

Number.	Braided Shapes	Thickness (mm)	Number of Layers	Strand Structure (mm)	Number of Shares	Total Number of Copper Wires	Maximum Heat Transfer Capacity (w)
A_1_	Banding [11]	0.4	2	0.04 × 9	17	153	4.25
A_2_	Banding [11]	0.4	2	0.04 × 10	17	170	5
A_3_	Banding [11]	0.4	2	0.04 × 11	17	187	5.25
A_4_	Annular [51]	0.4	NA	NA	NA	96	3.6
B_1_	Unoxidized ring shape [40]	1	NA	0.05 × 6	NA	NA	17
B_2_	Annular [40]	1	NA	0.05 × 6	NA	NA	20
B_3_	Annular [52]	1	1	NA	NA	152	15
B_4_	Annular [52]	1	2	NA	NA	224	20
C_1_	Annular [41]	1.1	2	0.04 × 4 + 0.05 × 2	24	288	18
C_2_	Annular [41]	1.1	2	0.04 × 5 + 0.05 × 2	24	336	24
C_3_	Annular [41]	1.1	3	0.04 × 6	24	432	26
C_4_	Annular [41]	1.1	2	0.04 × 7	24	336	13
C_5_	Annular [41]	1.1	2	0.05 × 7	24	336	15
D_1_	Unoxidized ring shape [40]	1.2	NA	0.06 × 6	NA	NA	20
D_2_	Annular [40]	1.2	NA	0.05 × 6	NA	NA	29

**Figure 9:**
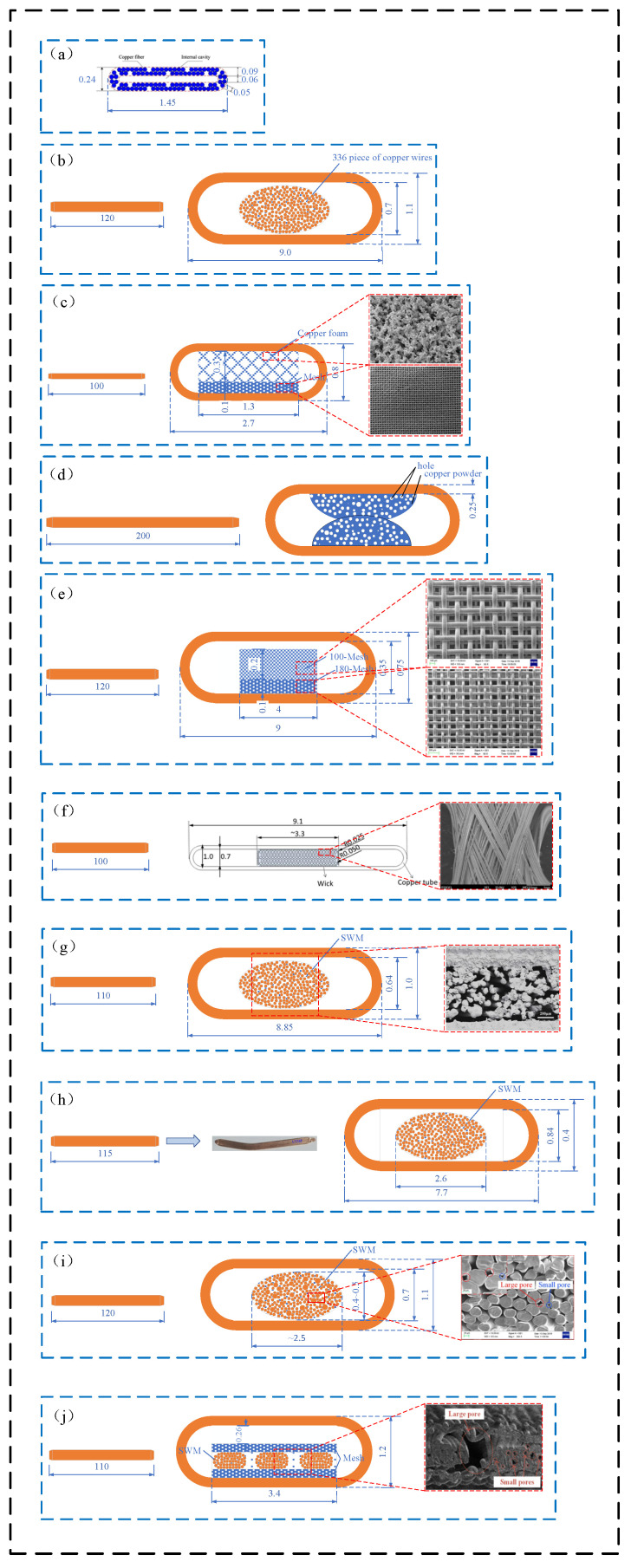
Schematic diagram, cross-sectional, and SEM images view of the internal structure of different ultra-thin flat heat pipes. SWM heat pipe: (**a**) [50], (**b**) [18], (**g**) [40], (**h**) [11]; composite wick heat pipe: (**c**) [45], (**e**) [55], (**f**) [52], (**i**) [41], (**j**) [56]; copper powder wick heat pipe: (**d**) [48] (unit: mm).

**Figure 10:**
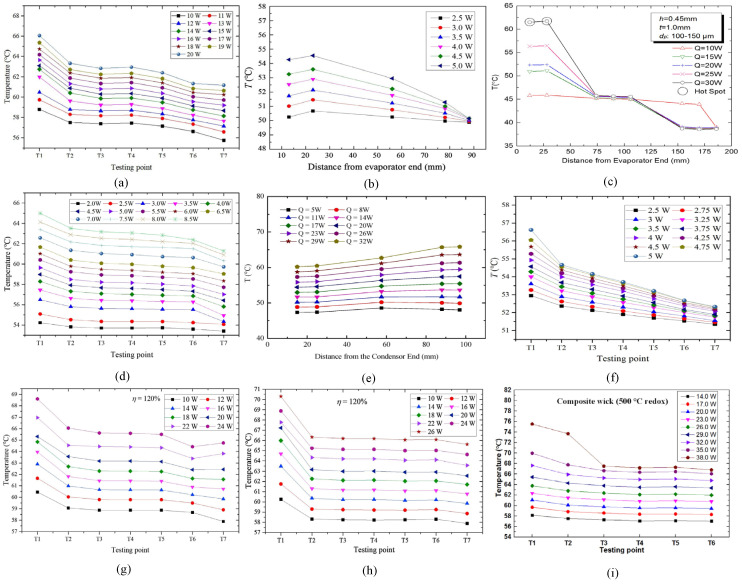
The temperature of each test point along the axial direction of the ultra-thin heat pipe of different literatures under different heating power, and the test points are uniformly distributed from the evaporation end to the condensation end of the heat pipe: (**a**) [18], (**d**) [55], (**f**) [11], (**g**) [41], (**h**) [41], (**i**) [56]; the test points are distributed along the distance from the evaporation end to the condensation end of the heat pipe: (**b**) [45], (**c**) [48], (**e**) [40].

Based on the structure and data of the ultra-thin flat heat pipe, ten similar articles are summarized to facilitate the comparative analysis of the factors affecting the heat transfer of the heat pipe. The structure is shown in Figure 9 [11,18,40,41,45,48,50,52,55,56], and the corresponding temperature profile is shown in Figure 10 [11,18,40,41,45,48,55,56]. Relying on the copper pipe as the carrier, the various derivatives (copper foam, mesh, spiral woven mesh, copper powder) are used to build wick structures, striving to achieve better heat transfer performance in small spaces.

The structure of wire mesh + SWM + wire mesh (Figure 9j [56]) can achieve the maximum heat transfer capacity of 38 W (Figure 10i [56]), and the temperature difference between the evaporation end and the condensation end is nearly 10 °C [56]. The heat transfer effect is more significant under high heating power [56]. Wire mesh can effectively retain pores when in the flattening process, and it has the advantages of a strong compression flattening ability and stable structure [56]. SWM makes the reflux resistance of condensate small, and it makes the vapor–liquid circulation more smooth [56]. The combination of wire mesh and SWM effectively solves the problem of excessive heat resistance between SWM and copper tube, and redox further improves its heat transfer performance [56]. However, it is difficult for this structure to function at the extreme ultra-thin thickness. On the one hand, it is the cost problem caused by the precise processing technology; on the other hand, the internal collapse of the SWM during extreme compression causes the porosity to be too low.

Comparing the temperature profile of b and f in Figure 10 [11,45], the maximum heat transfer capacity and temperature difference are both 5 W and 4 °C [11,45]. The performance of the structure in Figure 9h [11] is significantly better than that of the structure in Figure 9c [45] at low heating power. However, the overall temperature of the structure in Figure 9c [45] is lower than the structure in Figure 9h [11] when the heat pipe is in steady state operation. In terms of the overall size of the heat pipe, the structure in Figure 9c [45] and the structure in Figure 9h [11] are roughly the same. The flattened thickness of the structure in Figure 9c [45] is twice that of the structure in Figure 9h [11], but the width of the heat pipe of the structure in Figure 9h [11] is 285.2% of that of the structure in Figure 9c [45]. In the structure of the wick, the structure in Figure 9c [45] uses foamed copper mixed with mesh, and the structure in Figure 9h [11] is SWM for a single structure. Overall, the heat transfer performance of the two is roughly the same, but the processing cost of the structure in Figure 9h [11] is better than that of the structure in Figure 9c [45] due to its single structure, and its practicability is wider because of the 0.4 mm flattening thickness.

Comparing the structure in Figure 9c [45] and the structure in Figure 9e [55] with similar wick structures, the internal space of the structure in Figure 9e [55], of the heat pipe, is about three times that of the structure in Figure 9c [45], and the maximum heat transfer capacity of the structure in Figure 9e [55] is 170% of the structure in Figure 9c [45], as shown in Figure 10d [55] and Figure 10b [45]. The advantage in the internal space of the heat pipe does not bring ideal heat transfer to the structure in Figure 9e [55], and the structure of copper + mesh (Figure 9c [45]) is smaller than the mixing of meshes of different meshes.

Compared to the temperature profiles in Figure 10a [18], Figure 10g [41], and Figure 10h [41], both heat pipes have the same shell size and type of wick, and the only difference is that the composition ratio of the strands is slightly different during weaving. The overall temperature of the heat pipe in steady state operation is roughly the same. The maximum heat transfer capacity is 20 W, 24 W, and 26 W, respectively, and the temperature difference is 5 °C, 3 °C, and 5 °C [18,41]. It is proven that, by adjusting the number of strands, the heat transfer performance of the heat pipe can be slightly improved.

Compared with the structures in Figure 9d [48], Figure 9g [40], and Figure 9j [56], the structure of a double half-moon type (Figure 9d [48]), based on the mixing of copper powder with different meshes, also has a good performance, but the heat pipe of 200 mm length does not improve heat transfer performance. The axial temperature distribution of other heat pipes also shows that the length of the heat pipe has little effect on the heat transfer performance [40,48,56]. In the heat pipe, with a flattened thickness greater than 1.1 mm, the copper powder wick heat pipe has a lower production cost than the SWM heat pipe. In addition, it has a larger application with the heat transfer performance of excellence [48]. The data of the structure in Figure 9g [40] and the structure in Figure 9j [56] are similar in all aspects. From the structure in Figure 9j [56], it can be seen that the combination of the mesh and the SWM is not regular, and the contact of the joint parts is not regular. In addition, thermal resistance limits its heat transfer performance [56].

### 2.3. Formation of Ultra-Thin Heat Pipes

The head and tail of the heat pipe need to be necked (the vacuum pumping end is proposed to be the tail). In order to facilitate the welding and sealing of ultra-thin heat pipe and the cold welding of the second degassing process, the necking taper is matched with the front end of the mandrel to prevent the leakage of wick material [57]. In addition, the local pressure drop, caused by the sudden change of pipe diameter, is also conducive to protecting the working fluid in the heat pipe [57]. Conventional necking processes include rotary forging, extrusion, spinning, and direct punching [57]. As spinning and direct punching cause greater damage to the pipe, affecting the subsequent forming of the heat pipe, ultra-thin heat pipe mainly uses rotary forging and extrusion of two radial forging neckings [57].

Rotary forging necking is a process in which the hot pipe is continuously radially forging the outer wall of the hot pipe, by a high-speed rotating forging die, to reduce the diameter [57,58]. The forming force of the intermittent radial forging process is small, so it is suitable for thin-walled and large reduction ratios of tubing reduction [58]. For an ultra-thin hot pipe with thin wall thickness, the necking process is easy to spin off and cause deformation of the pipe body, so it is needed to reduce the spindle speed and adjust the rotary forging necking die [59]. Rotary forging has high requirements on the precision and impact resistance of the mold, and the required equipment is complex and precise [59]. In addition, the operating cost of rotary forging is high, and there is a lot of noise during the operation. [59].

The extrusion neck process is that the forming die rotates and extrudes the copper pipe, which is driven by the electric spindle [60]. The heat generated by rotating friction can reduce the deformation resistance of the copper pipe, but it can also cause oxidation of the copper pipe [57]. The extrusion neck has low dependence on the overall mold and does not require high precision, so the process cost is low [57]. Wang [60] et al. verified the results through experimental analysis and simulation with ABAQUS finite element software, which showed that the optimal necking process parameters for copper pipes, with outer diameter of 5 mm and wall thicknesses of 0.1, 0.2 and 0.3 mm, were a die feed rate of 50 mm/s and die speed of 4800 r/min.

The sintering process is important in the heat pipe manufacturing process, which is based on the principle of the diffusion bonding of metals at high temperatures. Sintering enables the formation of the wick and the combination of the wick and the pipe wall to achieve a certain bond strength and porosity to enhance the heat transfer capability of the heat pipe. Metal powder sintering is formed by the surface diffusion of the sintered neck, while metal fibers and wire mesh also need to diffuse through the volume and grain boundaries [61]. The sintering process is sequentially divided into four stages: reduction, sintering, holding, and cooling [53]. After a series of comprehensive analyses, Li et al. [61] found that the sintering temperature of 140~170 μm diameter copper powder was generally controlled at about 900~950 °C, holding for about 30~60 min, and the various properties of the prepared wick were better. While the sintering temperature of copper fiber and copper wire is usually lower than that of copper powder [61], the sintering neck becomes larger at too high of a sintering temperature, causing the pores to decrease, thus increasing the bonding strength of the wick and the difficulty of drawing the mandrel [61]. Copper powder sintering is more adequate than copper fiber sintering, which is an important reason for the higher capillary capacity of copper powder sintered wick [61].

**Figure 11:**
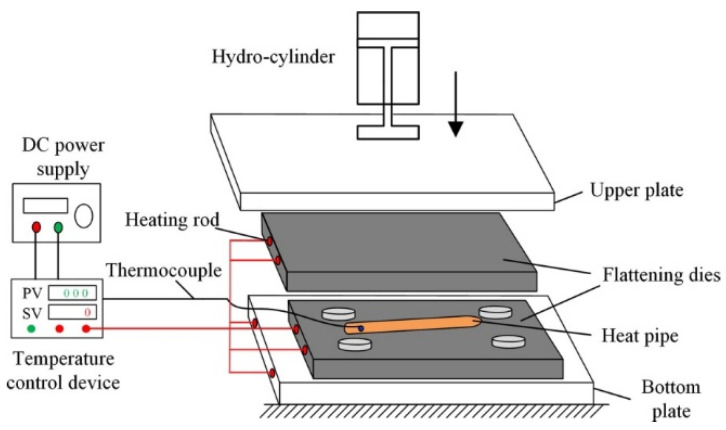
Schematic diagram of the phase-change flattening process [47].

The phase change flattening process, which is very important for the forming of ultra-thin heat pipes, was developed by Jiang et al. [54], based on the principle of steam pressure of the working fluid inside the heat pipe, to overcome the buckling phenomenon. The principle of the phase change flattening process is shown in Figure 11 [47], and Li et al. [47] pointed out that this process is more flexible and convenient than the welding process, as well as more suitable for industrial mass production. Using stamping die to compress the heat pipe as the core of its process, obvious elastic-plastic deformation occurs during the stamping process, and the vapor pressure in the tube needs to be kept constant during the stamping process [47]. In order to ensure that the ultra-thin heat pipe does not buckle during the phase change flattening process and does not rupture and scrap due to excessive vapor pressure, Tang et al. [62] combined the Von Mises yield quasi with the stress analysis of the phase change flattening process, and through repeated experimental tests, the optimal heating temperature for the phase change flattening of ultra-thin heat pipes was derived to be 220 °C.

### 2.4. Surface Modification

In the gas–liquid phase change heat transfer process of heat pipes, pool boiling is the main means of dissipating the larger heat fluxes [63]. Changes in both surface structure wettability and roughness affect the contact effect between solid and liquid. Thus, the state of boiling heat transfer was changed [64]. The hydrophilic surface can effectively improve the surface wettability and facilitate the flow of liquid to nucleation sites [65], while the heat transfer contact area and the number of nucleation sites increase [66]. In addition, the hydrophilic surface lead to the turbulence intensity and heat transfer coefficient of the working fluid in the pipe increasing [67], and it can successfully eliminate the inhibitory effect of bubble separation due to the network of connected micropores on the surface of the wick [68]. Studies have shown that modifying the surface of the heat pipe wick into a super-hydrophilic surface has a positive effect on the heat transfer performance of the heat pipe. The formula, based on the maximum capillary pressure ∆*P_c_*_,*max*_ of the wick, can also confirm this view. ∆*P_c_*_,*max*_ can be obtained from the Young–Laplace formula [69]:

(1) $$∆P_{c,max}=\frac{2\sigma cos\theta }{r_{eff}}$$

Here, *σ* is the surface tension of the working liquid; *θ* is the contact angle of the working liquid; *r_eff_* is the effective capillary radius of the wick. From the equation, it can be concluded that, when the effective capillary radius of the wick is determined, the maximum capillary pressure is only determined by the surface tension and contact angle, and the maximum capillary pressure of the wick can be effectively increased when the wick is changed to a superhydrophilic surface by surface modification.

**Figure 12:**
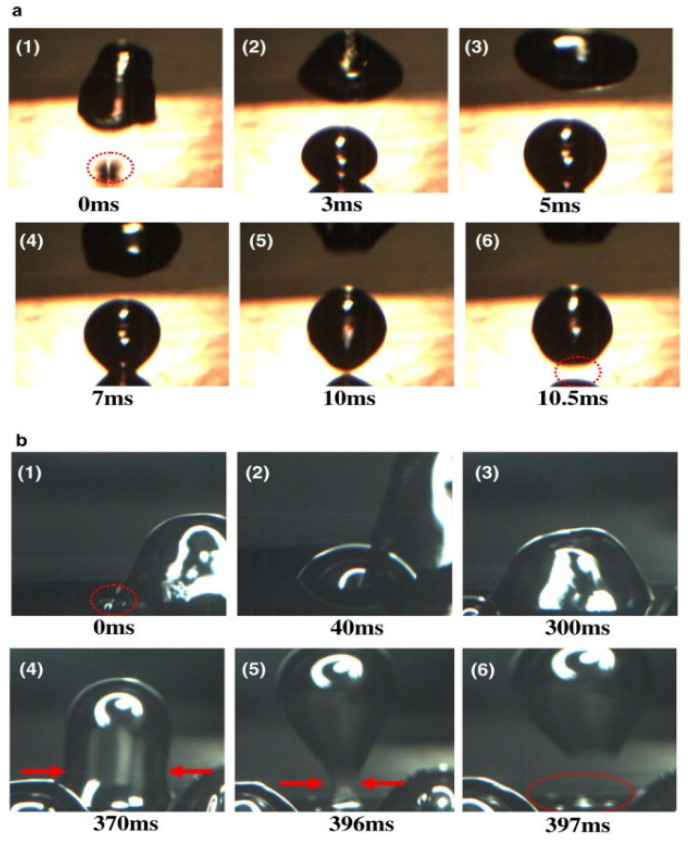
Generation process of bubbles: (**a**) hydrophilic surface; (**b**) hydrophobic surface [71].

To investigate the pool boiling heat transfer properties, foam metal was used as the experimental object, and the foam metal surfaces were hydrophilically and hydrophobically modified. Meanwhile, foam metal without surface modification was added as the reference group [70]. The experimental data show that the hydrophobic surface has higher heat transfer performance at low heat flux conditions [70]. However, the heat transfer performance of the hydrophilic surface gradually improved with increasing heat flux, and the best heat transfer performance was achieved when the heat flux exceeded 4 × 10^5^ W/m^2^ [70]. In addition, the reinforcement effect of low porosity hydrophilic surfaces is better than that of hydrophobic surfaces [70]. To further investigate the effect of surface structure wettability on boiling heat transfer, Jo et al. [71] analyzed the boiling heat transfer phenomena of different wettability surfaces (hydrophilic surface, hydrophobic surface, and heterogeneous wettable surface composed of a hydrophilic surface with hydrophobic dots) using surface modification techniques of microelectromechanical systems (MEMS) and high-speed visualization techniques. As shown in Figure 12 [71], (1) to (6) show the boiling heat transfer phenomena at different times, and it can be seen that hydrophilic surfaces are more favorable for the generation and separation of bubbles. However, when the contact angle of the material surface is greater than 90°, the direction of the force changes and inhibits the contraction of the bottom of the bubble when it separates from the surface [72]. Therefore, the contraction of the bottom of the bubble, on a hydrophobic surface, is subject to greater resistance [72].

Zhuo et al. [65] conducted experiments with untreated hydrophilic silicon wafers and super hydrophilic silicon wafers treated by silicon dioxide deposition. The experimental results show that the hydrophilic surface appears with a drying phenomenon under the condition of high heat flux, which leads to the decline of heat transfer performance [65]. However, there is no drying on the super hydrophilic surface, the pressure drop is stable, and the heat transfer performance is more stable because there is no deposited silica, and the wettability of the super hydrophilic surface is better [65].

All of the above studies are based on the effect of surface modification on boiling heat transfer, and it is well demonstrated that modifying the surface of heat pipe wicks to superhydrophilic surfaces has a positive effect on the heat transfer performance of heat pipes. Low capillary capacity has been a key factor hindering the development of screen sintered wick heat pipes, and the application of superhydrophobic modification, in the field of heat pipes, can significantly improve the hydrophilic performance of screen wicks and improve the capillary capacity. In addition, for the superhydrophobic modification, Tang [28] used the superhydrophobic surface with low surface energy in the condensing section to make it difficult for the condensate droplets to maintain the water droplet shape and, thus, keep the droplet condensation, which effectively reduces the thermal resistance and improves the heat transfer performance.

**Figure 13:**
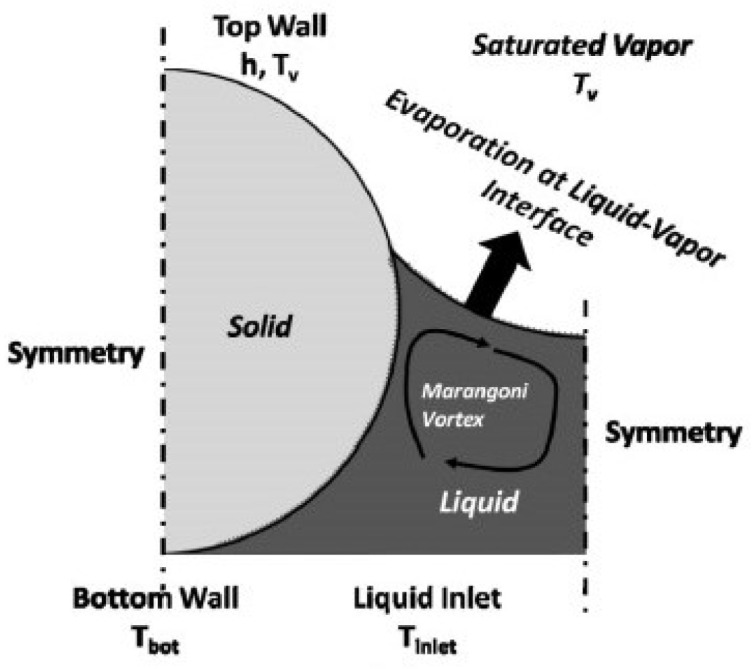
Thin film curved liquid surface at the solid–liquid interface and its boundary conditions [74].

Because of the high coupling inside the heat pipe, surface modification affects the vapor–liquid contact region and solid–liquid contact region inside the heat pipe, and the thermodynamic phenomena generated in the vapor–liquid contact region and solid–liquid contact region inside the heat pipe have been systematically analyzed by developing transient and steady-state models [73,74]. Harmand et al. [73] proposed a transient model that can capture the thermodynamic cycle inside the heat pipe. This model couples a 3D transient thermal model with a 2D hydrodynamic model based on mass conservation equations, analyzes the importance of heat transfer in the microfilm region at the vapor–liquid interface, and can be used to predict the avoidance of local hot spots using simulations [73]. Ranjan et al. [74] used a 3D numerical model of heat pipe transients to analyze the effect of contact angle and porosity on the mass flux in the pores of the liquid wick, and they found that the change in mass flux is inversely proportional to contact angle and porosity due to the fact that it is the solid-liquid interface. A thin film curved liquid surface that can enhance the convection effect in a small way is formed at the contact interface, as shown in Figure 13 [74], and the curved liquid surface keeps shrinking when the contact angle and porosity increase, which leads to the decrease in mass flux.

In order to study the motion law of the vapor–liquid phase change inside the heat pipe more in-depth, Fang et al. [75] proposed a three-dimensional liquid–gas phase change lattice Boltzmann model, which can operate in complex transient environments and effectively overcome the challenges posed by coupled heat transfer inside the heat pipe, as well as eliminate the assumptions that the wick must be saturated and that the porous wick structure is a continuous medium in previous studies and can simulate any wick structure [75]. It is found that, as the heat flux rises, the working fluid retreats into the groove wick and forms a curved meniscus at the solid–liquid interface, forming a pressure difference with the flat meniscus at the condensing end to obtain sufficient capillary force [75].

**Figure 14:**
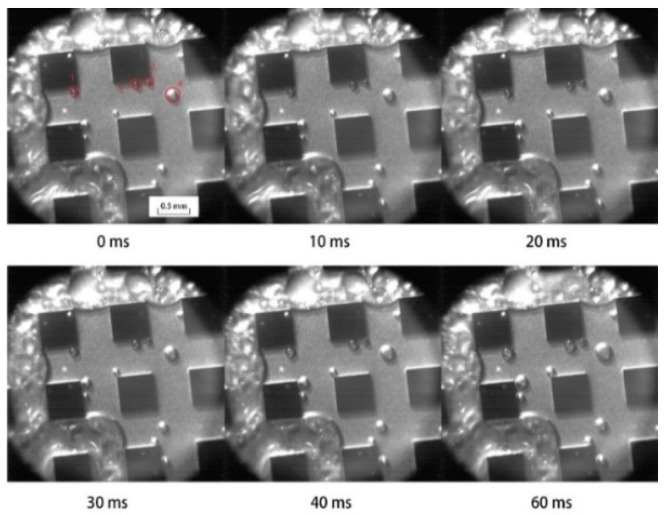
The process of bubble generation [76].

In the heat transfer process of the heat pipe, the heat transfer performance of the heat pipe and the internal vapor–liquid phase change process are inextricably linked. Cui et al. [76] investigated the bubble growth process in the flat region of the wick by Mixture model and experiment, and they deduced that the bubble growth rate is proportional to the heat flux and the surface area of the wick. Therefore, increasing the surface area and surface roughness of the wick has a positive effect on the heat transfer of the heat pipe [76]. As shown in Figure 14 [76], nanoscale nucleated bubbles are generated on the solid–liquid surface and gradually form a vapor film, which then eventually becomes millimeter-sized bubbles by separating and merging.

### 2.5. Novel Manufacturing Process

**Figure 15:**
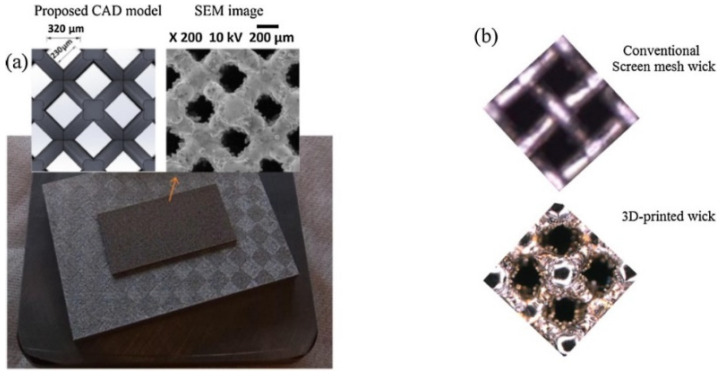
CAD model and SEM images for different manufacturing processes [79]: (**a**) additively manufactured wicks; (**b**) conventional wicks.

Many researchers have made new attempts to improve the manufacturing process of heat pipes. Shioga et al. [77] designed a flat loop heat pipe for small and thin electronic devices with a thickness of only 0.6 mm. Six chemically etched copper sheets with a thickness of 0.1 mm were used to superimpose them by heating and pressing [77]. This novel manufacturing method eliminates the gap between the inner wall of the evaporator and the wick, thus inhibiting vapor leakage and improving thermal leakage [77]. Feng et al. [78] used a flame spraying process to inject copper powder and aluminum powder into a flame spray gun at the same time, and then, they sprayed it onto the copper substrate covered with aluminum mesh. The aluminum is removed by sodium hydroxide to form a porous copper wick, and the porosity is determined by the feeding rate of the aluminum powder [78]. This novel fabrication method helps to reduce the production cost and simplify the manufacturing process for application in commercial manufacturing production [78]. Jafari et al. [79] applied additive manufacturing techniques to flexibly control the porosity and pore size, as well as the shape and size, of the wicking wick to fabricate a flat heat pipe with better heat transfer performance, using stainless steel as the raw material, as shown in Figure 15 [79]. When Zhou et al. [13] designed an ultra-thin heat pipe for telephone watches, the shrinkage process and annealing process at the head end was omitted from the conventional heat pipe manufacturing process, which effectively saved cost and improved production efficiency.

## 3. Heat Transfer Mechanism

The main function of the wick in an ultra-thin heat pipe is to provide capillary pressure to the condensate, thus the wick helps working fluid to return quickly to the evaporator and participate again in the vapor–liquid cycle. Therefore, the capillary force and permeability of the capillary pipe are the most important factors affecting the thermal performance of the ultra-thin heat pipe. For vapor flow in a heat pipe, Cotter [69] assumes that the axial flow of vapor is incompressible laminar flow and uses the radial Reynolds number to determine the radial flow of vapor, which is defined by the equation [69]:

(2) Rer=12πμvdmvdx

Here, *m_v_* is the mass flow rate of the steam; *μ_v_* is the viscosity of the steam [69]. The working fluid flow in the heat pipe is stable and close to the wall, and the Reynolds number is small when combined with the formula, so the working fluid flow state is laminar flow. When the capillary pressure of the capillary structure is greater than the flow resistance, the ultra-thin heat pipe can operate normally. Based on the laminar flow and incompressibility of the working fluid, the pressure balance equation inside the ultra-thin heat pipe, under normal conditions, is obtained as follows [8]:

(3) $$∆P_{c,max}\geq ∆P_{l}+∆P_{v}+∆P_{evp}+∆P_{con}\pm ∆P_{g}$$

Here, ∆*P_l_* is the working liquid flow pressure drop in the wick from the condenser to the evaporator section; ∆*P_v_* is the total vapor flow pressure drop in the ultra-thin heat pipe; ∆*P_evp_* is the interface pressure drops due to vapor; ∆*P_con_* is the interface pressure drops due to condensation; ∆*P_g_* is the gravitational pressure drop [8]. ∆*P_evp_* and ∆*P_con_* are usually negligible, especially under small thermal load conditions [8].

Based on the above analysis, the capillary limit of the ultra-thin heat pipe *Q′_max_* of the ultra-thin heat pipe can be obtained as [69]:

(4) $$Q_{max}^{\prime }=\frac{2\sigma cos\theta }{r_{eff}L_{eff}}{\left[\frac{8\mu_{v}}{\pi \rho_{v}r_{v}^{4}h_{lv}}+\frac{\mu_{l}}{K\rho_{v}A_{w}h_{lv}}\right]}^{-1}=\frac{2\sigma cos\theta }{r_{eff}L_{eff}}{\left[\frac{32\mu_{v}}{\rho_{v}D_{h}^{2}A_{v}h_{lv}}+\frac{\mu_{l}}{K\rho_{l}A_{w}h_{lv}}\right]}^{-1}$$

Here, *μ_v_* is the vapor dynamic viscosity; *ρ_v_* is the vapor density; *r_v_* is the radius of the vapor space; *A_v_* is the vapor passage area; *h_lv_* is the latent heat of the liquid; *L_eff_* is the effective length of the ultra-thin heat pipe; *D_h_* is the hydraulic diameter of the ultra-thin heat pipe [69]. The maximum heat transfer of the ultra-thin heat pipe is determined by the wick suction limit (*Q′_max_*) at a given operating temperature [69]. The axial heat transfer of the heat pipe relies, mainly, on convective heat transfer, which is all done by the process of vapor condensation and the release of latent heat in the condensing section [69].

The above vapor flow pressure drop ∆*P_v_* is derived from the cumulative pressure drop in the evaporator section, the adiabatic section, and the condenser section, and Li et al. [80] derive the equation for the flow pressure drop in the evaporator based on Darcy’s law:

(5) $$∆P_{w}=\frac{\mu_{l}\cdot {\bar{\mu }}_{l,w}\delta_{w}}{K_{p}}$$

**Figure 16:**
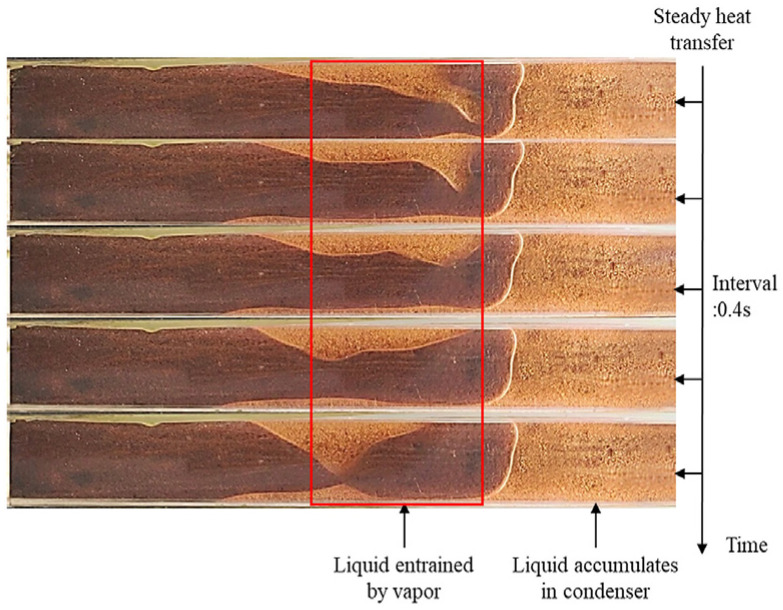
Vapor–liquid entrainment phenomenon [81].

Here, *μ_l_*_,*w*_ and *δ_w_* are the magnetic permeability [80]. When the heat flux is high and the gaseous working medium in the heat pipe is in a high-speed state, vapor–liquid entrainment may be caused [81]. The gaseous working medium entrains part of the liquid working medium to the condensing end, causing the accumulation of liquid working medium at the condensing end [81]. The thermal resistance also rises rapidly, hindering the normal operation of the heat pipe, thereby affecting the heat transfer performance of the heat pipe, as shown in Figure 16 [81]. Li et al. [82], based on the heat pipe theory proposed by Cotter and taking into account the vapor-liquid, determined that the entrained friction, resulting from the reverse motion and the buoyancy force on the water vapor under the influence of gravity and temperature difference conditions, was refined and modified as follows:

(6) $$∆P_{buoy,v}+∆P_{c,max}\geq ∆P_{l}+∆P_{v}+∆P_{evp}+∆P_{con}+∆P_{entrainment}\pm ∆P_{g}$$

Here, ∆*P_buoy_*_,*v*_ is the buoyancy force exerted on the water vapor by the temperature difference between the evaporating and condensing sections under gravity; ∆*P_entrainment_* is the additional pressure drop caused by the shear stress, generated by the liquid–vapor counterflow, at the interface of the two phases [82]:

(7) $$∆P_{entrainment}\approx \xi \cdot ∆P_{v}$$

**Figure 17:**
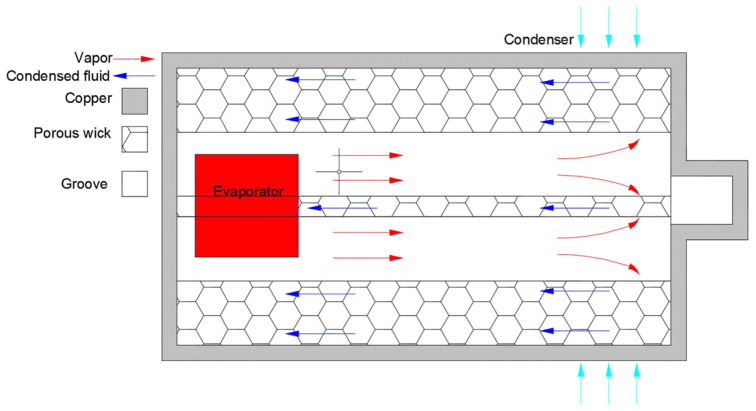
Mechanism of operation of a fluted flat heat pipe [83].

Here, ξ is the ratio of wetted to non-wetted surfaces at the boundary of the wick (for a saturated wick ξ = 1) [82]. In order to prevent the vapor–liquid entrainment phenomenon and overcome the shear stress caused by the liquid–vapor counterflow, Zhu et al. [83] separated the vapor–liquid channel of the heat pipe, and the gaseous mass used a groove as a separate channel, effectively avoiding the losses caused by the two phase masses, and the operation mechanism is shown in Figure 17 [83].

**Figure 18:**
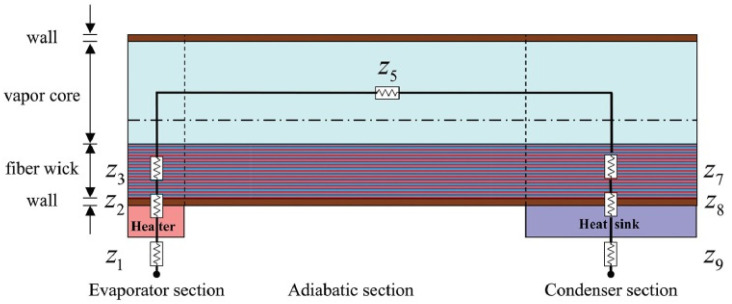
Traditional heat pipe equivalent thermal resistance network [84].

In order to gain a deeper understanding of the heat transfer process inside the heat pipe, researchers have analyzed the factors influencing the heat transfer efficiency of the heat pipe, based on the study of thermal resistance. The thermal resistance analysis of the heat pipe includes the steady state and transient state [84]. The thermal resistance analysis diagram can be obtained based on the circuit diagram and satisfies the series–parallel rule, as shown in Figure 18 [84], and the thermal resistance network method is usually used for thermal resistance analysis, based on Fourier’s law of heat transfer.

The thermal resistance *R* is obtained according to the Fourier law of heat conduction [85]:

(8) $$R=\frac{L}{kA}$$

**Figure 19:**
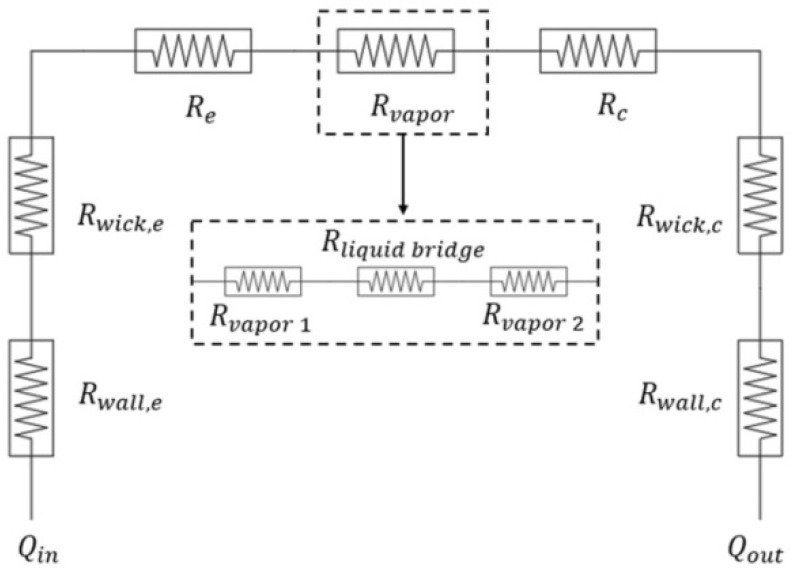
Thermal resistance equivalent network in the presence of liquid bridge [86].

Here, *L* is the heat transfer length; *k* is the thermal conductivity; *A* is the heat transfer area [85]. Based on the Claperon equation, Li et al. [86] further analyzed the thermal resistance *R_vapor_* of the evaporator section. When a liquid bridge occurs in the evaporator section, an additional heat transfer resistance *R_liquidbridge_* needs to be added, and the specific thermal resistance network is shown in Figure 19 [86].

In order to analyze the heat pipe thermal resistance more precisely, Li et al. [87] added the radial thermal conductivity thermal resistance *R*_1_, *R*_2_, and the thermal resistance *R*_3_, generated by the phase change heat transfer at the vapor–liquid interface, in the evaporation section of the heat pipe. In addition, the thermal resistance *R*_4_, generated by the axial flow of steam and heat transfer, is added [87].

The above thermal resistance analysis is for a planar section in the steady state case of the heat pipe. For the thermal resistance of the cylinder in the transient condition of the heat pipe, Li et al. [87] also performed a complete theoretical derivation and simulation, and they obtained the governing equations for each thermal resistance block by analyzing a single thermal resistance.

As shown in Table 6 [86,87], the thermal resistance of the evaporator section is relatively complicated. The liquid bridge and the vapor–liquid interface of the phase change heat transfer will hinder heat conduction [86]. In addition, the heat will generate radial heat conduction thermal resistance when passing through the wick and the heat pipe wall, respectively [87]. The heat inside the heat pipe is conducted in accordance with Fourier’s law, the axial part is mainly conducted through gaseous and liquid working fluids, and the axial thermal resistance along the wick and the heat pipe wall can be ignored [87].

## 4. The Factors affecting Heat Transfer Performance and Optimal Design

Many factors affect the heat transfer performance of ultra-thin heat pipes, which can be divided into internal and external factors. Internal factors mainly refer to the properties of the heat pipe itself, including the aforementioned wick structure, as well as the filling rate and working fluid type, while external factors refer to the external environment when the heat pipe is applied. Both internal and external factors have different degrees of influence on the heat transfer performance of the heat pipe. The heat pipe not only needs a suitable manufacturing process and an excellent wick structure but also needs to be reasonably tuned to various parameters and optimally designed through various optimization methods. In this section, the factors affecting the heat transfer performance and the optimized design are briefly introduced and analyzed, and the heat transfer performance of heat pipes under different influencing factors is summarized.

The filling rate is an important factor affecting thermal resistance and thermal conductivity. Lips et al. [88] presented the combined effect of filling rate and vapor space thickness on the heat transfer performance of flat heat pipes. The results of the study showed that the variation of filling rate leads to the variation of thermal resistance, and either too high or too low filling rate results in very small heat transfer efficiency, thus an optimum filling rate exists [88]. In addition, the optimum fill rate for flat heat pipes with a vapor space thickness of 2 mm is between 10% and 25% [88]. Chen and Chou [89] also conducted a related study on fill rate, using acetone as the working fluid to investigate the effect of liquid fill rate on the thermal performance of flat heat pipes, and the experimental results showed that the correct fill rate plays a decisive role for flat heat pipes at higher input heating power.

The tilt angle is also critical to the thermal performance of the ultra-thin heat pipe. Changes in the tilt angle, combined with gravity, cause changes in the working fluid distribution and change the rate at which the working fluid flows back into the evaporator. The angle of tilt is positively correlated with the return velocity. Too low or too high of a return velocity can lead to a reduction in the efficiency of heat exchange at the condenser. Tharayil et al. [90] revealed that the tilt angle affects the thermal performance of the heat pipe and found that the wick has a positive resistance to gravity. The stability of the heat pipe self-start was also verified by iterative experiments [90]. Liu et al. [91] also explored the effect of tilt angle on heat pipe performance by tilting a miniature grooved heat pipe, where different working fluids are affected by the tilt angle to varying degrees, and in addition, the tilt angle affects the thermal resistance. In the mapping of the heat transfer performance of flat parallel-flow heat pipes, Shen et al. [32] found that the heat transfer performance of flat parallel-flow heat pipes was significantly enhanced at larger inclination angles. When studying multi-channel flat heat pipes, Guichet et al. [92] excavated the law of inclination angle and thermal resistance. At low heating power, the inclination angle has a great influence on the evaporation thermal resistance of the heat pipe, and it has little effect on the condensation thermal resistance, but with the gradual increase in heating power, the exact opposite occurs [92].

**Figure 20:**
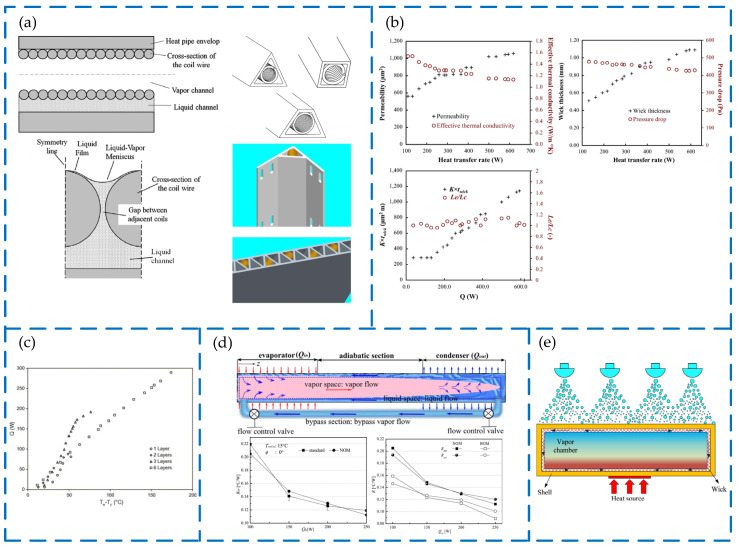
Different optimized designs: (**a**) schematic diagram of the spiral coil heat pipe structure and its application design [93]; (**b**) the relationship between heat flux, optimal wick permeability and effective thermal conductivity, heat flux, optimal wick thickness, as well as the relationship between the relevant pressure drop, heat flux, ratio of optimal evaporator length to condenser length (*Le/Lc*), and optimal wick permeability (*K*) and wick thickness (*t_wick_*) [94]; (**c**) the effect of the number of layers of wire mesh on the heat transfer rate of the heat pipe in the horizontal direction [95]; (**d**) The operation diagram of the heat pipe with auxiliary pipes, the thermal resistance comparison of standard heat pipe, NOM, and BOM, where *R_HP_* is heat pipe thermal resistance, *R_Sys_* is system thermal resistance [96]; (**e**) schematic diagram of the combination of heat pipe and spray cooling [98].

To further enhance the heat transfer performance of the heat pipe, researchers have optimally enhanced the heat pipe through different optimization methods. As shown in Figure 20a [93], Li used helical coils to flexibly match different heat pipe shells and found that the local lateral temperature difference in the heat pipe may be optimized under zero gravity. Jafari et al. [94] developed a mathematical model using the genetic algorithm II optimization method to optimize the structural parameters of the sieve wick with the design parameters as decision variables. The optimization result is shown in Figure 20b [94]. As shown in Figure 20c [95], Kempers et al. obtained heat pipes with different wick thicknesses by adjusting the number of wire mesh layers, and they found that the influence rate of the number of wire mesh layers on thermal resistance is low. The thermal resistance is only increased by 40% under six times the thickness of the wick, and the six-layer wire mesh greatly improves the maximum heat transfer capacity of the heat pipe [95]. In order to reduce the thermal resistance of the heat pipe, as shown in the Figure 20d [96], Baek et al. added an auxiliary pipe from the condensation end to the evaporation end on the basis of the conventional heat pipe to facilitate the return of the condensate to the evaporation end. When the heat pipe is placed horizontally, the thermal resistance of NOM (auxiliary channel off) is very close to the standard heat pipe of the same specification, but the thermal resistance of BOM (auxiliary channel on) is significantly smaller than that of NOM, which confirms the positive effect of developing auxiliary pipes on the performance of heat pipes [96]. Jung et al. [97] also conducted a similar optimization experiment. The maximum heat transfer rate of the heat pipe was increased by nearly 35.5%, under the condition of horizontal placement, but the optimization effect decreased under the inclined angle [97]. The optimal design of the heat pipe is more flexible, as shown in the Figure 20e [98], so Zhao et al. combined the heat pipe and spray cooling technology. Under the spray speed of 1.63 L/min, the thermal resistance of the heat pipe is 0.0469 K/W, and the corresponding effective thermal conductivity is as high as 2371.77 W/(m·K) [98]. The heat transfer performance of spray cooling technology is much higher than that of air-cooled radiators and water-cooled radiators [98].

In summary, the filling rate, tilt angle and working fluid all affect the heat transfer performance to different degrees, but because of the individual variability of ultra-thin heat pipes, there is a positive degree of error in the study, and it needs to be combined with the actual situation in the application process. The optimal combination of parameters can be gained by an analysis of the results from single variable experiments in an orderly grouping.

## 5. Conclusions and Prospect

In terms of preparation technology and heat transfer performance, the research status of ultra-thin heat pipes is reviewed and analyzed in this paper. The future development is also put forward based on the review of the research status of ultra-thin heat pipe at the present stage.

(1) With the development of thin heat pipe and the high requirement for heat transfer performance, the traditional manufacturing process of heat pipe makes it difficult to meet the development needs, so it is necessary to improve the traditional manufacturing process and develop a new manufacturing process. Taking super hydrophilic treatment as the representative, the combination of super hydrophilic treatment and spiral braiding technology realizes high heat conduction of heat pipe in the ultra-thin field.

(2) In terms of heat transfer performance, taking the spiral braided mesh wick as the wick research object and combining it with the experimental data, changing the copper wire diameter could not effectively solve the heat transfer failure of the ultra-thin heat pipe with low thickness, but the heat transfer performance could be improved by making different pores with a copper wire mixture. In addition, the heat transfer performance of the ribbon spiral woven mesh wick is better at the extreme ultra-thin thickness (less than 0.4 mm), and the influence of ultra-hydrophilic treatment on the heat transfer performance is reduced in this case.

(3) Two methods to solve the contact thermal resistance should be developed. The contact between the pipe shell and the wick is not absolutely tight, so there is a certain contact thermal resistance, which will directly affect the heat transfer performance of the ultra-thin heat pipe. It is necessary to develop an optimized method to reduce the contact thermal resistance. On the one hand, the pipe shell and the wick can be integrated. In order to enhance the mechanical properties, short copper wires are added to the copper powder, and then, the heat pipe integration can be realized by sintering and surface treatment technology. On the other hand, based on copper powder, mesh, and SWM, the composite wick structure is constructed under two flattening thicknesses of 0.3~0.5 mm and 0.6~1.0 mm, respectively, and the contact thermal resistance between the small-sized copper powder and the pipe wall is small. Combined with the advantages, the SWM closely fits the heat pipe shell and, then, wraps it with mesh to increase the overall mechanical properties of the wick, preventing the pores in the SWM from being too small when the flattening thickness is too low. A good ratio can achieve a better heat transfer effect.

(4) A systematic ultra-thin heat pipe system and standard should be established. The lack of systematic research on ultra-thin heat pipes, the lack of unified research standards among different researchers, and the lack of a mature system would hinder the development of ultra-thin heat pipes, to a certain extent, and slow down the progress of ultra-thin heat pipes. Given the higher requirements and challenges of ultra-thin heat pipes, measurement standards for heat pipes with different parameters should be formulated, and unified standards for experiments, theory, and simulation should be established. For each new type of heat pipe, the heat transfer performance of the heat pipe is tested from the capillary infrared rise height, the axial temperature distribution under different heat input, the evaporation heat resistance and condensation heat resistance, as well as the heat transfer capacity of different filling rates to facilitate the other researchers to conduct follow-up data comparison, obtain a better heat pipe structure, and compare the influencing factors, which determine each influencing factor of heat pipe.

## Figures and Tables

**Table 1:** Advantages and disadvantages of heat pipes with different shell materials and their application fields.

Materials	Advantages	Disadvantages	Application Scenarios	Literature
aluminum	Low cost of raw materials, light weight and simple processing	Poor compatibility, easy to be corroded, low thermal conductivity	Fields where heat pipe quality is important, such as aerospace	[19,22,23]
Stainless steel	Low cost of raw materials, high mechanical strength, strong corrosion resistance, high temperature resistance, good compatibility	Difficult to process, low thermal conductivity	In extreme environments, such as high temperature conditions, highly corrosive conditions, and fields requiring long service life	[21,24]
copper	High thermal conductivity, low processing difficulty, good compatibility, low processing cost	High cost of raw materials and high material density	Heat transfer requires large fields, such as electronic appliances, communications, solar energy	[11,12,13,14,15,16,17]

**Table 2:** TP1 optical pipe properties [27,28].

Tubing Parameters	Numerical Value
Cu Purity (%)	99.97
Density *ρ*_s_ (Kg/m^3^)	8.96 × 10^3^
Thermal conductivity *k*_s_ (J/m·K)	393
Modulus of elasticity *E* (GPa)	117.8
Yield strength σ_s_ (Mpa)	227
Hardness (HV)	75–120
Melting point (°C)	1080
Poisson’s ratio ν	0.33

**Table 3:** Working fluids at different working temperatures [29,30,31].

Temp. Range	Specification
>700 K	Using liquid metals such as potassium, sodium and silver, very high heat fluxes can be obtained due to the inherent properties of the fluid, namely very large surface tension and high latent heat of vaporization.
550–700 K	Using specialty organic fluids such as naphthalene and biphenyl.
200–550 K	Using water, ammonia, acetone, methanol and ethanol etc.
1–200 K	With working fluids such as helium, argon, neon, nitrogen, and oxygen. Using helium, argon, neon, nitrogen and oxygen etc. Due to the very low latent heat of vaporization value and the low surface tension of the working fluid, they generally have relatively low heat transfer capabilities.

**Table 4:** Maximum heat transfer capacity of ultra-thin heat pipe with different wick structures and thickness.

Wick Type	Thickness (mm)	Maximum Heat Transfer Capacity (w)	Literature
Copper fiber wick	0.35–0.6	5	[43]
Central fiber wick	0.4	1.5	[43]
Grooved wick	0.6	7.2	[44]
Copper foam wick	0.8	5	[45]
Silk screen wick	1	6–11	[46]
Single arch sintered channel wick	1	12	[47]
Double-sided arched sintered channel wick	1	13	[47]
Reticulated fluted wick	1	14	[47]
Copper powder wick	1	20	[40]

**Table 6:** Summary of different types of thermal resistance.

Thermal Resistance Type	Formulas	Key Parameters
The thermal resistance of the evaporator section *R_vapor_* [86]	$R_{vapor}=\frac{12R\mu_{v}T_{v}^{3}L_{eff}}{\rho_{v}P_{v}t_{v}^{3}h_{fg}^{3}w}$	
Additional thermal resistance when liquid bridges occur in the evaporator *R_liquidbridge_* [86]	$R_{liquidbridge}=\frac{L_{bridge}}{k_{wall}A}$	*L_bridge_* is the length of the liquid bridge; *k_wall_* is the thermal conductivity of the heat pipe wall;
Radial heat conduction and thermal resistance of pipe wall of evaporation section *R*_1_ [87]	R1=ln(d0di)2πλwL1	*d_0_* is the outer diameter of the heat pipe; *d_i_* is the inner diameter of the heat pipe; *λ_w_* is the thermal conductivity of the pipe wall; *L*_1_ is the length of the evaporation section
Radial heat conduction and thermal resistance of the wick in the evaporation section *R*_2_ [87]	R2=ln(didv)2πλeL1	*d_v_* is the diameter of the vapor space inside the pipe; *λ_e_* is the thermal conductivity of the wick compound
The phase change heat transfer thermal resistance at the vapor–liquid interface in the evaporation section *R*_3_ [87]	$R_{3}=\frac{RT_{v}^{2}\sqrt{2\pi RT_{v}}}{r^{2}p\pi d_{v}L_{1}}$	*R* is the vapor gas constant; *T_v_* is the vapor temperature; *r* is the latent heat of vaporization; *p* is the vapor pressure
Thermal resistance caused by steam axial flow heat transfer *R*_4_ [87]	$R_{4}=\frac{128L_{eq}\mu_{v}T_{v}}{\pi d_{v}^{4}\rho_{v}^{2}r}$	*L_eq_* is the effective length of the heat pipe; *μ_v_* is the viscosity of the steam; *ρ_v_* is the vapor density
The governing equation of each thermal resistance block under the transient condition of the heat pipe [87]	$\frac{dT_{i}}{dt}=\frac{2\alpha_{i}}{\lambda_{i}^{2}}\left(T_{i,1}+T_{i,2}-2T_{i}\right)$	*T_i_* is the central temperature of the thermal resist block; *T_i_*_,1_ and *T_i_*_,2_ are the boundary temperatures on both sides of the thermal resist block, *λ_i_* is the thermal conductivity thickness of the thermal resist block

## Nomenclature

*ρ*_s_	Density (Kg/m^3^)
*k*_s_	Thermal conductivity (J/m·K)
*E*	Modulus of elasticity (GPa)
σ_s_	Yield strength (Mpa)
ν	Poisson’s ratio
*t*	heat pipe flattening thickness
*dp*	copper powder particle size
*h*	maximum thickness of the absorbent wick
∆*P_c_*_,*max*_	maximum capillary pressure of the wick
*σ*	surface tension of the working liquid
*θ*	contact angle of the working liquid
*r_eff_*	effective capillary radius of the wick
Re*_r_*	radial Reynolds number
*m_v_*	mass flow rate of the steam
*μ_v_*	viscosity of the steam
∆*P_l_*	working liquid flow pressure drop in the wick from the condenser to the evaporator section
∆*P_v_*	total vapor flow pressure drop in the ultra-thin heat pipe
∆*P_evp_*	interface pressure drops due to vapor
∆*P_con_*	interface pressure drops due to condensation
∆*P_g_*	gravitational pressure drop
*Q*′*_max_*	capillary limit of the ultra-thin heat pipe
*ρ_v_*	vapor density
*r_v_*	radius of the vapor space
*A_v_*	vapor passage area
*h_lv_*	latent heat of the liquid
*L_eff_*	effective length of the ultra-thin heat pipe
*D_h_*	hydraulic diameter of the ultra-thin heat pipe
∆*P_w_*	flow pressure drop in the evaporator
*μ_l_*_,*w*_	magnetic permeability
∆*P_buoy_*_,*v*_	buoyancy force exerted on the water vapor by the temperature difference between the evaporating and condensing sections under gravity
∆*P_entrainment_*	additional pressure drop caused by the shear stress generated by the liquid-vapor counterflow at the interface of the two phases
ξ	ratio of wetted to non-wetted surfaces at the boundary of the wick
*L*	heat transfer length
*k*	thermal conductivity
*A*	heat transfer area
*R_vapor_*	thermal resistance of evaporator section
*R* *_liquidbridge_*	additional thermal resistance when liquid bridges occur in the evaporator
*R*_1_	radial heat conduction and thermal resistance of pipe wall of evaporation section
*R*_2_	radial heat conduction and thermal resistance of the wick in the evaporation section
*R*_3_	phase change heat transfer thermal resistance at the vapor-liquid interface in the evaporation section
*R*_4_	thermal resistance caused by steam axial flow heat transfer
*L_bridge_*	length of the liquid bridge
*k_wall_*	thermal conductivity of the heat pipe wall
*d_0_*	outer diameter of the heat pipe
*d_i_*	inner diameter of the heat pipe
*λ_w_*	thermal conductivity of the pipe wall
*L*_1_	length of the evaporation section
*d_v_*	diameter of the vapor space inside the pipe
*λ_e_*	thermal conductivity of the wick compound
*R*	vapor gas constant
*T_v_*	vapor temperature
*r*	latent heat of vaporization
*p*	vapor pressure
*L_eq_*	effective length of the heat pipe
*T_i_*	central temperature of the thermal resist block
*λ_i_*	thermal conductivity thickness of the thermal resist block
